# Soft skills for medical physicists: Evolving a profession

**DOI:** 10.1002/acm2.70531

**Published:** 2026-03-05

**Authors:** Tomas Kron, Julie Raffi, Shada Wadi‐Ramahi, Abdelkader Toutaoui, Bilal Jalal, Peter A. Sandwall, Graciela Velez, Dario Sanz, Godfrey Azangwe

**Affiliations:** ^1^ Peter MacCallum Cancer Centre and Sir Peter MacCallum Department of Oncology Melbourne University Melbourne Victoria Australia; ^2^ Centre for Medical Radiation Physics University of Wollongong Wollongong New South Wales Australia; ^3^ Department of Radiation Oncology Duke University School of Medicine Durham North Carolina USA; ^4^ Department of Radiation Oncology University of Pittsburgh School of Medicine and UPMC Hillman Cancer Center Pittsburgh Pennsylvania USA; ^5^ Department of Radiation Oncology and Molecular Imaging Hôpital Chahids Mahmoudi Tizi Ouzou Algeria; ^6^ Johns Hopkins Aramco Healthcare (JHAH) Dhahran Saudi Arabia; ^7^ King's College London (KCL) London UK; ^8^ Department of Radiation Oncology Ohio Health Mansfield Hospital Mansfield Ohio USA; ^9^ Dosimetry and Medical Radiation Physics Section, Division of Human Health, Department of Nuclear Sciences and Applications International Atomic Energy Agency Vienna Austria

**Keywords:** education of medical physicists, Professionalism

## Abstract

**Purpose:**

Medical physicists are essential healthcare professionals who bridge the gap between technology and patient care, particularly in radiation oncology and medical imaging. With the profession expanding its clinical and global roles, the need for competencies beyond technical expertise—such as communication, leadership, and cultural awareness—is increasingly evident. These competencies, commonly referred to as soft skills, are critical for patient‐centered care but remain insufficiently integrated into formal education and training pathways. The aim of the present work was to identify soft skills relevant to medical physics practice and investigate where in a career they are acquired and used.

**Methods:**

This paper presents the views of a group of medical physicists affiliated with leading organizations in medical physics education and professional development. The group conducted a comprehensive analysis of the role and relevance of soft skills in clinical practice, academic settings, and international training programs. Their discussions led to the identification, classification, and mapping of essential soft skills across different career stages and professional roles within the field. The findings aim to inform curriculum development, professional standards, and capacity‐building initiatives in medical physics worldwide.

**Results:**

A framework of core soft skills was developed and categorized into seven domains: professionalism, leadership, cultural/political awareness, communication, adaptability, emotional intelligence, and ethical reasoning. These skills were mapped to various career stages of medical physicists, from university coursework to clinical practice and international expert missions. The analysis demonstrated that soft skills are dynamic, teachable, and essential across academic, clinical, and global contexts. The study also reviewed current gaps and opportunities in integrating soft skills into medical physics curricula, clinical residency programs, and continuing professional development.

**Conclusion:**

To meet the evolving demands of healthcare, soft skills may need to be embedded in the education, training, and professional development of medical physicists. These skills enhance interdisciplinary collaboration, patient engagement, and leadership capacity, positioning medical physicists as integral members of the healthcare team. Academic institutions, professional societies, and global organizations are encouraged to work together to define, teach, and assess these competencies in ways that are practical and culturally adaptable.

## 1 Introduction

According to the International Labor Organization (ILO) ‘medical physicists are considered to be an integral part of the health work force alongside those occupations classified in sub‐major group 22, health professionals’ even if they are listed as part of ‘Physicists and Astronomers.’^1^ This duality between physical science and clinical service shapes many aspects of the profession and makes medical physics a challenging career that demands broad education and training. It also allows physicists to address complex problems based on a deep understanding of physics from first principles and its application to medical technology. Their role includes ensuring the safe and effective use of radiation, as well as contributing to broader areas such as advanced computing and medical imaging beyond ionizing radiation.

However, with increasing demands and broadening professional scope, it is becoming clear that existing training and education frameworks fall short in cultivating the full range of competencies related to soft skills. While technical expertise remains essential, it is no longer sufficient. In practice, physicists are routinely required to make decisions that directly affect patient care, such as providing dosimetric comparisons and quality improvement input to support clinical implementation. Soft skills are essential for medical physicists—not only in their daily clinical responsibilities but also in broader roles such as teaching internationally, participating in audits, and serving as international experts or researchers. These situations require clear communication alongside ethical judgment while considering environmental and cultural factors—skills that are rarely taught, cultivated, or assessed during formal training. The absence of soft skills^2^ in education leaves many physicists ill‐equipped to fulfill their clinical role with confidence, potentially compromising patient safety and treatment effectiveness and weakening interdisciplinary and international collaboration. In contrast, the value of soft skills for physicians, nurses and physician assistants have been shown to improve patient experience and save lives.^3^, ^4^, ^5^, ^6^, ^7^

While recent papers and professional society efforts have increased awareness of the importance of soft skills, their systematic integration into established education and training frameworks remains limited.^8^, ^9^, ^10^ There is a clear need to embed these competencies directly within the medical physics training pathway. Hence, academic institutions, professional societies, and international organizations have both the opportunity and responsibility to make efforts to provide resources and frameworks for soft skills development. The question of assessing and evaluating these acquired skills, either as part of academic or clinical training, poses a challenge. Is it viable to reciprocate the experiences of other healthcare professionals, such as in medical and nursing education,^11^, ^12^, ^13^ or do medical physicists need a unique evaluation metric?

This paper presents the collective vision of medical physicists convened at a consultancy meeting organized by the International Atomic Energy Agency (IAEA) with participation of physicists from North America, Africa, Asia, Australia and South America in Vienna (April 1–4, 2025). The experts for the consultancy meeting were appointed by the IAEA based on their demonstrated clinical and technical expertise, as well as their experience in delivering education and training in international settings. Care was taken to ensure balanced regional representation. Experts were expected to be aware of international literature and documents and bring in relevant local experience.

The objective of this consultancy meeting was to draft guidelines for soft skills required for lecturers, trainers, auditors and experts in medical physics, particularly in the context of international training activities. These guidelines are aimed at complementing other publications by professional societies and the IAEA which cover some of the elements, for example the (AAPM) TG 159^14^ and the IAEA TCS 78^15^ on guidelines on professional ethics for medical physicists.

The scope was purposely extended to consider soft skills not only for international trainers but also for practicing clinical medical physicists. It stands to reason that the skills of auditors, trainers, and experts—such as cultural awareness, conflict resolution, advocacy, and leadership—may also be relevant to clinical physicists. Yet, the traditional focus on scientific rigor above all else may come at the expense of developing interpersonal, ethical and organizational competencies. This document aims to define soft skills relevant for medical physicists and discuss where soft skills could be acquired and applied within a medical physics career. Based on this analysis we are providing a vision for medical physics in the future.

## 2 Soft skills and medical physics

The term ‘soft skills’ is used to describe personal attributes and values often intertwined with emotional intelligence, such as communication, adaptability, ethical reasoning, and leadership. These skills enable professionals to collaborate effectively, advocate for safety, and resolve conflicts in complex interdisciplinary environments. Effective communication is essential for interactions with colleagues, administrators, patients and the public.^16^ The premise of this article is that soft skills can be taught or at least acquired in a structured way. As a result, soft skills may be included in curricula and assessed. Soft skills can be divided into several often‐overlapping groups:

- Universally needed soft skills that enhance interpersonal interaction
- Soft skills that are contingent on the culture or society in which the professional operates
- Relevant soft skills for a particular profession.

A list of soft skills relevant to medical physics is shown in Table 1. All tables were developed using brainstorming with iterative refinement. A second meeting of the group was held during the International Conference on Advances in Radiation Oncology (ICARO‐4) in June 2025 to review the material.

**TABLE 1:** Attempt at identifying, categorizing and sorting soft skills relevant for medical physicists.

Category	Soft skills/attributes/values	Comment
**Professionalism**	Integrity; Confidence in role; Teamwork; Impartiality; Accountability; Time management	Core attributes underpinning credibility and reliability.
**Leadership**	Conflict management; Diplomacy; Team building; Advocacy; Role modeling	Essential for guiding teams and shaping organizational culture.
**Cultural and political awareness**	Respect for diversity; Language awareness; Diplomacy; Advocacy; Awareness of hierarchy and power dynamics; Social dynamics	Social dynamics include gender, class, and professional hierarchies that influence practice.
**Communication**	Active listening; Interpersonal skills; Interprofessional communication; Conflict resolution; Negotiation; Patient/public speaking; Providing and receiving feedback; Technical communication; Scientific writing; Time management	Central to collaboration with colleagues, patients, and the public.
**Adaptability/Flexibility**	Resilience; Creativity; Problem‐solving; Acceptance of disagreement	Enables responsiveness to changing environments and viewpoints.
**Emotional intelligence**	Empathy; Humility; Grace; Moderation; Flexibility; Perseverance	Supports collaboration, mentorship, and patient interaction.
**Ethical reasoning**	Patient safety focus; Acknowledgment of limitations; Owning mistakes; Ethical practice; Holistic thinking	Anchors decision‐making across all other soft skills.

It is important to note that Table 1 is not exhaustive in respect to soft skills for medical physicists. Other skills may be needed and the grouping of skills into seven broad categories is a choice of the authors.

Providing a definitive list of soft skills may be impossible as there is overlap and interdependence between the skills. The choice of inclusion depends on cultural context and language, with soft skills grouped and prioritized differently depending on the setting. The inclusion of ethics in the list illustrates this approach. This entry does not prescribe a particular ethical framework, but emphasizes the ability to apply ethical principles transparently, consistently, and with sound reasoning. Regardless of the ethical framework applied, ensuring patient safety should be a consistent anchor point for all practicing medical physicists.^15^

There are other abilities that are sometimes included in the definition of soft skills, such as marketing, organizational politics, and project management. Project management is an example of a skill that can be learned and trained. It even is used to provide a ‘job description’ and while it requires several of the soft skills listed, does not in itself constitute a personal attribute that underlies the ability of professionals to interact effectively with others.

## 3 Soft skills throughout the medical physics career

Soft skills are not static. While there is a core set of skills that can be identified (Table 1), their importance shifts with career stage and cultural context. The soft skills required by professionals also vary throughout their career and there are many publications in the context of healthcare.^17^, ^18^

Table 2 provides a structured view of how soft skills can be mapped across different stages in a medical physicist's career and highlights the evolving nature of these skills, from academic training and supervised research to clinical practice and expert missions. While the specific emphasis may vary across cultural or institutional contexts, the table reinforces the idea that soft skills are not static traits, but dynamic competencies cultivated intentionally throughout a physicist's professional journey. Notably, trainers and auditors must be prepared to apply nearly all categories of soft skills. Supplementary material to the manuscript provides more details on this specific topic as it was the focus of the expert meeting.

**TABLE 2:** Selected soft skills mapped against different phases in a medical physics career.

Skill	University coursework	Supervised research	Clinical training	Professional practice	Supervision and management	Continuing professional development	Mentorship	Needed for a trainer/auditor/expert mission
Professionalism	✓	✓	✓	✓	✓	✓	Possible	✓
Accountability	✓	✓	✓	✓	✓	–	Possible	✓
Teamwork	✓	✓	✓	✓	✓	–	Possible	✓
Leadership	–	–	✓	✓	Possible	–	✓	✓
Advocacy	–	–	✓	✓	✓	Possible	✓	✓
Integrity	✓	✓	✓	✓	✓	✓	Possible	✓
Cultural/Political Awareness	✓	Possible	✓	✓	✓	Possible	Possible	✓
Communication	✓	✓	✓	✓	✓	✓	✓	✓
Adaptability/Flexibility	–	✓	✓	✓	–	✓	✓	✓
Emotional intelligence	–	–	✓	✓	Possible	–	✓	✓
Ethical reasoning	✓	✓	✓	✓	✓	✓	✓	✓

## 4 Inclusion of soft skills in training and education programs

An increasing number of medical physics training programs are taking soft skills into account. This applies to academic education as well as clinical training programs. In academic and clinical training, ideally at the postgraduate level, soft skills are often included under the broad category of ‘professionalism’ which covers leadership, ethics, and communication.^19^, ^20^ It should be noted that without clear definitions endorsed by professional societies, explicit soft skills training is often neglected. Without guidance on how to teach these skills it is easy to reduce the teaching of these non‐technical skills to reading assignments only. The same would apply to assessments.^21^ AAPM's TG 159^14^ presented a recommended ethics curriculum for medical physics graduate and residency programs in 2010. The IAEA produced a guidance document, IAEA TCS 78^15^ on Professional Ethics for Medical Physicists after a survey which was conducted in 2021 revealed a lack of understanding of professional ethics and a need for training, educational resources and continuous professional development activities on this topic in most settings. More recently, examples of curricula for foundational patient‐centered skills have been published and can serve as a resource to help other institutions develop their own programs.^21^ Similar guidance is needed for some of the other soft skills identified in Table 1. More so, there is a clear need for an objective assessment and evaluation metric to guide learners during their efforts of acquiring a skill. While there is general guidance in respect to soft skill assessments,^22^ publications focusing on medical physics are rare.^21^

Many guidelines for clinical training of medical physicists refer to soft skills.^23^, ^24^, ^25^, ^26^ However, experienced trainers themselves are less likely to have had soft skills explicitly included in their training. It is interesting to note the change over time which can be seen by comparing four Guidelines for Hospital Based Medical Physics Residency Training Programs published by the AAPM from 1990 to 2024.^23^, ^27^, ^28^, ^29^

Clinical training programs for medical physicists are expected to be two to three years in length after graduate level education is completed.^23^, ^24^, ^25^ It would be difficult to extend the length of clinical training to include new subjects so the inclusion of soft skills training must be balanced with existing training priorities and timelines.

Most training programs lead to an examination and certification.^30^ The International Medical Physics Certification Board (IMPCB) does note a few soft skills but the focus is more weighted toward practical skills directly related to the medical physicist's role in the clinic. (https://www.impcbdb.org/exams/exam‐preparations/requirements10b/) The American Board of Radiology (ABR) now also includes content on Professionalism and Ethics with a recommended series of online modules hosted by the Radiological Society of North America (RSNA; (https://education.rsna.org/Users/ChefViewCatalog.aspx?Criteria=120&Option=665&SearchMode=1). Yet there is still a lack of guidance on the teaching and assessment of such skills.

It is important to note that soft skills are part of a lifelong learning process and as such will also appear in mentorship programs^31^, ^32^ as well as continuing professional development (CPD).^30^, ^33^

## 5 Soft skills in Research and Development

Many medical physicists would consider research and development part of their professional role.^33^, ^34^ Effective communication, collaboration, and leadership are essential for translating scientific ideas into impactful outcomes. Soft skills are integral to many of these activities. These soft skills include the ability to articulate research findings clearly, work across disciplinary boundaries, and guide project teams through complex problem‐solving processes. Specific soft skills relevant to research include scientific writing, teamwork, leadership, adaptability, critical thinking, and ethical reasoning. Ethical reasoning remains critical for navigating questions of authorship, data ownership, and research integrity—especially in global and multi‐institutional studies.^2^, ^34^, ^35^

## 6 Soft skills as an indicator for the changing role of medical physicists

The increasing emphasis of soft skills in medical physics practice highlights a change in the role of medical physicists. Soft skills are now integral in all subspecialties of medical physics and bring the different specialties closer together. Professional needs across disciplines are increasingly overlapping; for example, there is an increasing need for radiotherapy physicists to work with images seen in Image Guided Radiation Therapy (IGRT)^36^ as well as imaging physicists to oversee therapies. The latter is particularly visible in the emerging field of theranostics.^37^, ^38^

**FIGURE 1:**
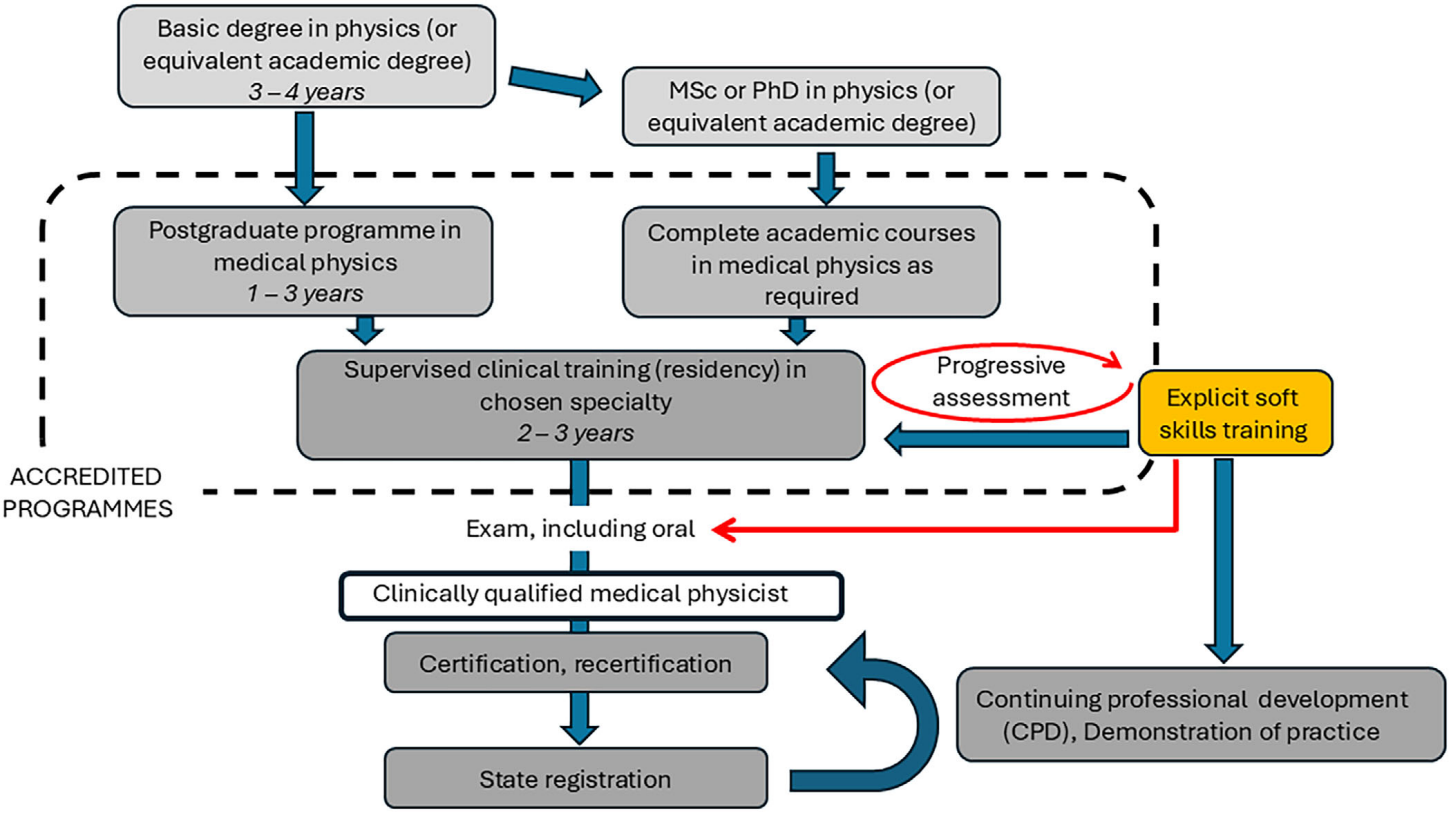
Training and career pathway for clinical medical physicists adapted from the IAEA schematic^30^, ^42^ to include consideration of soft skills.

As medical physicists grow in their career, they become more involved in mentorship, training and global outreach. Medical physicists are increasingly called upon to support education and capacity‐building efforts worldwide, particularly in underserved regions.^39^, ^40^, ^41^ These responsibilities demand soft skills such as cultural intelligence, diplomacy, and adaptive communication. It may therefore be appropriate to expand the schematic training program developed by the IAEA^30^, ^42^ as shown in Figure 1 to explicitly include soft skills.

Figure 1 indicates that soft skills training is—at least initially—not part of many accredited training programs. However, an assessment may be appropriate in particular as the trainee progresses into a clinical role. The figure highlights that this may be conducted as part of a progressive assessment.^43^ The figure also proposes including soft skills into the ongoing education program for medical physicists. This is relevant because it also allows practicing physicists to acquire formal training for soft skills even if this was not part of their own training. However, it is not necessarily an easy task as CPD activities should be easy to document and verify.^30^ Soft skills training should ideally be longitudinal and competency‐based across academic education (MSc/PhD), clinical training, and certification stages.

Given the broad scope of soft skills, they also provide an opportunity for medical physicists to specialize. The traditional subspecialties of medical physics (radiotherapy, radiology and nuclear medicine) are likely to require similar soft skills and the acquisition of specific skills such as patient communication can enhance the role of medical physicists in the clinic.^44^, ^45^ On the other hand, communication with the public could benefit someone in a more administrative or radiation protection role.^46^ One may even propose using soft skills to prepare medical physicists for different paths be it clinical, technical or managerial. There is no doubt that possessing appropriate soft skills will make medical physicists better academics, educators and leaders.

## 7 Conclusion

Including soft skills in medical physics education and practice complements the technical competencies. As medical physicists increasingly engage in direct patient care, interdisciplinary collaboration, international training, and healthcare leadership, their effectiveness depends not only on technical expertise but also on interpersonal, ethical, and communication competencies. Embedding soft skills systematically into academic programs, clinical residencies, and CPD would strengthen the profession's visibility, and align it with patient‐centered care models. While challenges remain in defining, teaching, and assessing soft skills across diverse cultures and healthcare systems, these obstacles are surmountable with coordinated efforts by professional societies, and international organizations.

Clinical medical physicists are more than technical experts; they are well‐rounded professionals and influential members of the healthcare team, who need soft skills to lead and collaborate with excellence.

## AUTHOR CONTRIBUTIONS

Godfrey Azangwe: Writing, coordination and review. Bilal Jalal: Writing and review. Tomas Kron: Concept, coordination and writing. Julie Raffi: Writing and review. Peter A Sandwall: Writing and review. Dario Sanz: Writing and review. Abdelkader Toutaoui: Writing and review. Graciela Velez: Writing and review. Shada Wadi‐Ramahi: Writing and review.

## CONFLICT OF INTEREST STATEMENT

Tomas Kron: Research collaboration agreement with Varian Medical Systems. Shada Wadi‐Ramahi: Associate editor of JACMP.

## Supporting information



Supporting Information
