# Supplementary material for: Soft skills for medical physicists: Evolving a profession
Source: J Appl Clin Med Phys. 2026 Mar 5;27(3):e70531. doi: 10.1002/acm2.70531 (PMC12961229; doi:10.1002/acm2.70531)
Supplement: Supplementary file 1 — Supporting Information [file ACM2-27-e70531-s001.docx]

**Supplementary material**

The specific task of the working group was to put together recommendations regarding soft skills, in a case study focused document, required by auditors and experts in missions organized by IAEA and other professional societies specifically as they apply to medical physicists. The following is a summary of these considerations:

**Medical Physicists as Facilitators in Training**

In medical physics education, facilitators play a crucial role in imparting technical knowledge, fostering engagement, and guiding the next generation of professionals. Successful training demands not only a deep understanding of physics and its clinical applications but also strong communication, leadership, and adaptability skills.

Effective Communication and Knowledge Transfer

Key Communication Skills:

- Clear presentation : Providing structured and straightforward explanations while steering clear of complex jargon.
- Active listening: Fostering participant involvement and addressing their requirements.
- Flexibility : Modifying teaching methods according to the audience's background and understanding.
- Non-verbal communication : Employing body language, visual tools, and demonstrations to enhance the learning experience.

Facilitating Group Engagement and Learning

Key Engagement Strategies:

- Encouraging dialogue : Posing open-ended questions to promote analytical thinking.
- Experiential learning : Implementing practical demonstrations, role-playing activities, and case studies.
- Equitable engagement : Making certain that every trainee has the opportunity to participate while moderating more vocal individuals.

Leadership and Mentorship in Training

Key Leadership Skills:

- Motivating learners : Fostering a motivating educational atmosphere that promotes ongoing development.
- Productive feedback : Delivering specific, actionable insights regarding trainees strengths and areas for growth.
- Emotional intelligence: Recognizing diverse learning preferences and addressing trainees’ needs with compassion.

**Medical Physicists as Auditors**

Medical Physicists are often called upon for audits. This ranges from dosimetric audits to clinical audits that assess workflows and the entire treatment chain in radiation oncology and imaging protocols in radiology. Radiation safety is another important field where medical physicists are involved in audits. A successful auditor would need to have a large number of soft skills with critical thinking, flexibility and diplomacy being particularly relevant.

Critical Thinking and Objectivity in Audits

Key Analytical Skills:

- Structured assessment : Adhering to established audit procedures and checklists.
- Attention to detail : Identifying the gaps, even within effectively operating systems.
- Objective evaluation : Judging based on evidence and established criteria instead of personal beliefs.

Diplomacy and Conflict Management

Key Conflict Resolution Skills:

- Effective communication : Presenting results in a non-confrontational manner.
- Influence and negotiation : Motivating organizations to adopt necessary corrective measures.
- Cultural sensitivity : Recognizing variations in practices across institutions and regions.

Report Writing and Recommendations

Key Report Writing Skills:

- Streamlined documentation : Presenting findings in a structured format.
- Comprehensive viewpoint : Emphasizing both strengths and opportunities for enhancement.
- Practical suggestions : Offering concrete steps for addressing issues.

**Medical Physicists as Expert Assessors and Consultants**

Given the scientific basis of physics, medical physicists are often used as expert assessors and consultants. This is not only in response to an incident but often related to quality improvements. Cultural awareness, professionalism and project management skills would be a good starting point for the soft skills needed.

Cross-Cultural and Political Awareness

Key Cultural Adaptation Skills:

- Respect for local practices : Understanding the limitations of institutional frameworks and healthcare systems.
- Interdisciplinary communication : collaborating with hospital management, healthcare providers, and regulatory agencies.
- Awareness of ethical and political considerations : Addressing conflicts of interest and navigating bureaucratic obstacles.

Strategic Decision-Making and Influence

Key Leadership and Policy-Making Skills:

- Strategic problem resolution : Recognizing both urgent and underlying issues.
- Stakeholder collaboration : Partnering with governmental entities, global organizations, and professional associations.
- Ethical accountability : Weighing technical precision against patient safety and the practicality of institutional operations.

Crisis Management and Adaptability

Key Adaptability Skills:

- Handling uncertainty : Maintaining poise in stressful circumstances.
- Innovative problem-solving : Identifying alternative strategies when conventional methods are ineffective.
- Fostering trust : Developing credibility to secure collaboration from institutions.
